# Seroprevalence and risk factors for *Trypanosoma evansi*, the causative agent of surra, in the dromedary camel (*Camelus dromedarius*) population in Southeastern Algeria

**DOI:** 10.4102/ojvr.v87i1.1891

**Published:** 2020-12-21

**Authors:** Mohammed H. Benaissa, Nora Mimoune, Younes Bentria, Tahar Kernif, Abdelaziz Boukhelkhal, Curtis R. Youngs, Rachid Kaidi, Bernard Faye, Youcef Halis

**Affiliations:** 1 Scientific and Technical Research Centre for Arid Areas (CRSTRA), Biophysical Station, Nezla, Touggourt, Algeria; 2 National High School of Veterinary Medicine, Bab-Ezzouar, Algiers, Algeria; 3 Laboratoire d’Eco-épidémiologie Parasitaire et Génétique des Populations, Institut Pasteur d’Algérie, Algiers, Algeria; 4 Animal Science Department, Iowa State University, Ames, United States of America; 5 Institute of Veterinary Sciences, LBRA, University of Blida, Soumaa, Blida, Algeria; 6 UMR SELMET, CIRAD-ES, Montpellier, France

**Keywords:** seroprevalence, *Trypanosoma evansi*, Camelus dromedarius, camels, Algeria

## Abstract

Surra, caused by *Trypanosoma evansi*, is a re-emerging animal trypanosomosis, which is of special concern for camel-rearing regions of Africa and Asia. Surra decreases milk yield, lessens animal body condition score and reduces market value of exported animals resulting in substantial economic losses. A cross-sectional seroprevalence study of dromedary camels was conducted in Algeria, and major risk factors associated with infection were identified by collecting data on animal characteristics and herd management practices. The seroprevalence of *T. evansi* infection was determined in sera of 865 camels from 82 herds located in eastern Algeria using an antibody test (card agglutination test for Trypanosomiasis – CATT/*T. evansi*). Individual and herd seroprevalence were 49.5% and 73.2%, respectively, indicating substantial exposure of camels to *T. evansi* in the four districts studied. Five significant risk factors for *T. evansi* hemoparasite infection were identified: geographical area, herd size, husbandry system, accessibility to natural water sources and type of watering. There was no association between breed, sex or age with *T. evansi* infection. Results of this study provide baseline information that will be useful for launching control programmes in the region and potentially elsewhere.

## Introduction

Trypanosomiasis is a significant animal and human health problem worldwide. Human African trypanosomiasis (sleeping sickness) is caused by infestation with the flagellate protozoan *Trypanosoma brucei*, whereas animal trypanosomiasis is caused by different species and subspecies of *Trypanosoma* (*T. congolense, T. vivax, T. brucei brucei* and *T. brucei evansi*). Amongst all of the trypanosomiases, ‘surra’ (caused by *Trypanosoma brucei evansi*) has the widest host range and the broadest geographical distribution. It is transmitted mechanically by the bite of infected tabanid flies; Family: Tabanidae (Desquesnes et al. 2009); Genus: *Tabanus, Muscidae stomoxys, Haematopota, Lyperosia* spp. and *Chrysops* spp. (Luckins 1998), or infected vampire bats (Stoco et al. 2017). Transmission occasionally occurs iatrogenically through herd identification and vaccination procedures (Gutierrez et al. 2005), and oral transmission was confirmed recently in a mouse model (Mandal et al. 2017).

Trypanosomosis serves as an important constraint to camel production because it substantially reduces animal productivity, thus inducing economic losses. It is a major endemic disease problem throughout Central and South America, Africa and Asia (Gutierrez et al. 2000). Infection with *T. brucei evansi* (hereafter, *T. evansi*) reduces market value of exported animals, decreases milk yield and lessens animal body condition score, resulting in more than $223 million loss to the camel industry (Salah, Robertson & Mohamed 2015). In addition to causing the aforementioned losses, *T. evansi* is the most important cause of infectious abortions in camels in the Middle East and Africa (Boushaki et al. 2019; More et al. 2017). Camels with clinical disease can present progressive emaciation, severe anaemia, reproductive problems (because of infertility, abortions and stillbirths), and eventually death (Desquesnes et al. 2013).

In Algeria, *T. evansi* was first detected in 1903 in infected camels (Sergent & Sergent 1905). Since that time, few studies have been performed on livestock, and most were conducted in a limited geographical area with a limited number of samples (Benfodil et al. 2020; Bennoune et al. 2013; Boushaki et al. 2019); those studies concluded only that the hemoparasite can be detected in blood samples. Limited data are available currently concerning the epidemiology and distribution of the *T. evansi* parasite in Algeria. To gauge levels of endemic stability, it is important to assess the seroprevalence of *T. evansi* and to characterise local variations in risk factors.

To help fill the scientific knowledge gap regarding this important disease, the present study was conducted with the objectives of determining the seroprevalence of *T. evansi* and investigating potential risk factors for infection with *T. evansi* in the one-humped camel (Camelus dromedarius) population in eastern Algeria.

## Materials and methods

### Study area

**FIGURE 1:**
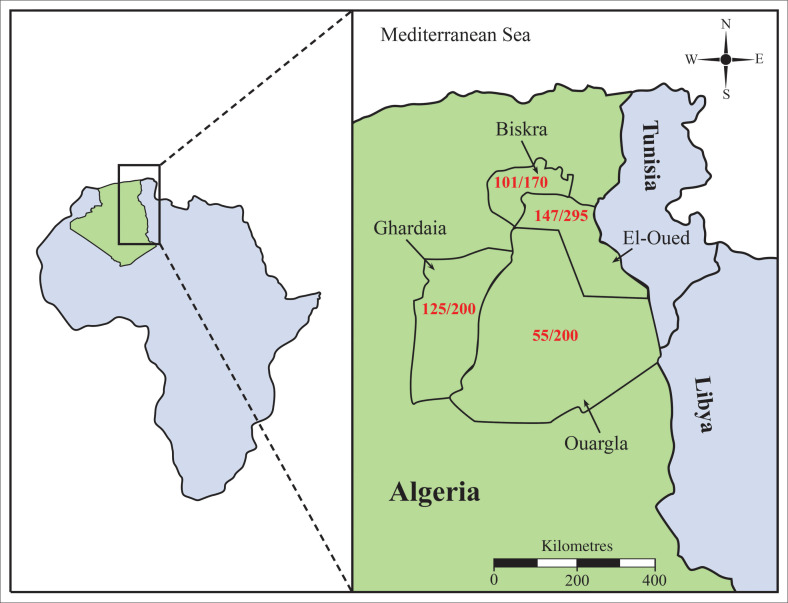
Distribution of *Trypanosoma evansi* seropositivity in dromedary camels in four districts in southeastern Algeria. Numbers in red indicate the number of camels seropositive for *Trypanosoma evansi* as a proportion of animals tested in the Biskra, El-Oued, Ghardaia and Ouargla districts.

This study was carried out in four districts (Biskra, El-Oued, Ouargla and Ghardaia) in eastern Algeria. These provinces are located at 00204–00735 E and 28°32–34°56 N (Figure 1). This region is considered as one of the most significant camel-rearing areas in Algeria, and it is a region where camel milk is increasingly commercialised and consumed. The climate of this region is arid and is characterised by long, hot summers and short, mild winters.

### Data collection

Data were collected using a paper-based questionnaire given to herd owners by trained investigators. The study questionnaire focused on 23 potential explanatory variables grouped by: (1) pastoralists’ socio-demographic characteristics (gender, age, occupation and formal education); (2) individual camel data (age class [< 1 year, 1–3 years, 4–9 years, 10–15 years and > 15 years; ages were assigned based on dental wear and owner information], sex [male, female], breed [Sahraoui, Tergui]); and (3) herd data (location [district], herd size [small: < 10 head, medium: 11–30 head, large: > 30 head], the introduction of new camels bought at a livestock market [yes or no], presence of other domestic animal species [sheep, goats, cattle; yes or no], epidemiological characteristics [occurrence of abortions in the herd], exchange of bulls for mating [yes or no] and animal management system).

### Sample size determination

The minimal sample size required for this study was determined using the random sampling method (Thrusfield 2018), considering an expected prevalence of 35% (based on unpublished survey data collected in 2008 in seven districts in Algeria) with 5% precision at the 95% confidence level. The formula used for calculation of sample size was:

$$N={\left(1.96\right)}^{2}\times \frac{P(1-P)}{d^{2}}$$ [Eqn 1]

where *N* = number of sample size, *P* = expected prevalence and *d*^2^ = absolute precision.

Although the minimum sample size calculated for this study was 350 animals, we increased the number of samples collected to improve the degree of accuracy and to account for some potential sample losses.

### Sample collection and processing

A total of 865 blood samples were collected in a randomised manner. A total of 10 mL of blood was collected by jugular venipuncture. Blood samples remained at room temperature for 3–5 h to allow clotting, followed by centrifugation for 15 min at 3000 revolution per minute (rpm). The serum fraction was extracted with a micropipette, and serum samples were frozen at −20 °C until analysis.

Sera were tested for the presence of anti-*T. evansi* antibodies using a commercially available direct card agglutination test (c*ard agglutination test* [CATT] for trypanosomiasis, *T. evansi* kit, Institute of Tropical Medicine, Antwerp, Belgium) according to the manufacturer’s instructions. Approximately, 45 *µL* of test reagent was transferred onto the test card and was mixed with an equal volume of the test sera diluted 1:4 with CATT buffer. The test card was rotated for 5 min at 70 rpm, after which time the reaction was checked carefully for agglutination. Positive reaction (evidence of agglutination) was determined by the quantitative presence of blue-colored granules (Bajyana-Songa & Hamers 1988). Individuals with a positive CATT-P titer _1:4 result (+ = weak, ++ = moderate, +++ = strong) were considered serologically positive.

### Statistical analysis

Pearson’s Chi-square test or Fisher’s exact probability test were applied to test for significant associations between potential risk factors and the outcome variable (status of *Trypanosoma* seropositivity in camels) in a univariate analysis. Multivariate logistic regression models were carried out using all variables showing a tendency towards statistical significance (*p* value £0.25). The logistic regression model was developed with a step-wise forward approach using a likelihood ratio test at each step with a *p* value of 0.10 as the significance level for removal or retention of the variable. In the final model, any variable with a *p* value < 0.05 was considered statistically significant. Model fit analysis was assessed with the Hosmer and Lemeshow goodness-of-fit test. All statistical analyses were performed using the Statistical Package for Social Sciences (SPSS) version 17.0 (SPSS Inc., Chicago, IL, United States [US]).

### Ethical consideration

Animal sampling was implemented with the approval of the national veterinary authorities and was exclusively done by veterinarians; the animals were blood-sampled without suffering and were subsequently released. Farmers in each zone gave verbal consent to participate in our study and gave permission for the blood sample collection from camels on their property. We followed the European guidelines (European directives EU 86/609-STE123 and 2010/63/EU) for animal handling.

## Results

### Individual level

Antibodies against *T. evansi* were detected in 428 of the 865 individual camels tested, giving an individual seroprevalence rate of 49.5% (95% confidence interval [CI]: 46.1% – 52.9%). At the herd level, 60 of the 82 herds tested had at least one seropositive animal, giving a herd seroprevalence rate of 73.2% (95% CI: 62.2% – 82.4%).

Results of the univariate analysis (Chi-square) revealed that individual animal seroprevalence for *T. evansi* was affected by geographical area, herd size, husbandry system, accessibility to natural water sources and type of watering (Table 1). Contrarily, no differences (*p* > 0.05) were demonstrated in trypanosomosis seroprevalence because of genetic sex, breed or animal age.

**TABLE 1:** Factors associated with animal-level seroprevalence of antibodies against *Trypanosoma evansi* for dromedary camel populations of southeastern Algeria.

Factor	Category	Number of animals	Serum *T. evansi* antibodies				p*
			Positive		Negative		
			*n*	%	*n*	%	
Geographic location (district)	Ouargla	200	55	27.5	145	72.5	0.000
	Biskra	170	101	59.4	69	40.6	
	El-Oued	295	147	49.8	148	50.2	
	Ghardaia	200	125	62.5	75	37.5	
Sex	Male	36	21	58.3	15	41.7	0.278
	Female	829	407	49.1	422	50.9	
Age class	1 (< 1 year)	7	4	57.1	3	42.9	0.158
	2 (1–3 years)	127	55	43.3	72	56.7	
	3 (4–9 years)	304	140	46.1	164	53.9	
	4 (10–15 years)	131	72	55.0	59	45.0	
	5 (> 15 years)	296	157	53.0	139	47.0	
Breed	Sahraoui	722	349	48.3	373	51.7	0.131
	Tergui	143	79	55.2	64	44.8	
Herd size	Large (> 50 head)	625	325	52.0	300	48.0	0.010
	Medium	159	75	47.2	84	52.8	
	Small (< 20 head)	81	28	34.6	53	65.4	
Husbandry system	Extensive	564	243	43.1	321	56.9	0.000
	Semi-intensive	150	100	66.7	50	33.3	
	Intensive	151	85	56.3	66	43.7	
Access to natural water sources	Yes	411	265	64.5	146	35.5	0.000
	No	454	163	38.9	291	64.1	
Watering system	Artificial wells	562	257	45.7	305	54.3	0.003
	Lakes and streams	303	171	56.4	132	43.6	

* , Univariate analyses (*χ*^2^ test for significance).

There was a very strong association between *T. evansi* seroprevalence and geographical area (*χ*^2^ = 4.80, *p* < 0.0001). The highest infection rate (62.5%, 125/200) was found in Ghardaia (northern part of the Sahara region), whereas the district of Ouargla had the lowest infection rate (27.5%, 55/200). The analysis showed that camels raised in large herds had a higher (*p* < 0.007) incidence of *T. evansi* infection compared with those raised in small herds.

Regarding the multivariate logistic regression analyses (Table 2), three variables remained in the final model: (1) herd size, (2) husbandry system and (3) water source. The odds of *T. evansi* infection were 2.2 times higher amongst large camel farms (*p* = 0.007, odds ratio [OR] = 2.159; 95% CI = 1262–3693) compared with small herds. Herds with more than 50 camels were 2.1 times more likely to have a seropositive animal than herds with fewer than 10 head. The presence of natural sources of water in nearby grazing areas was associated with an increased risk of *T. evansi* infection (OR = 9.415; 95% CI = 1.256–70, 583). Animals in a semi-intensive management system were more likely to be infected (OR = 2.95; 95% CI: 1.878–4.645) than extensively managed animals.

**TABLE 2:** Risk factors for individual-level infection with *Trypanosoma evansi* amongst dromedary camel populations in southeastern Algeria.

Factor	B	Std err	OR	95% CI (OR)	*p* value*
Constant	−1.028	0.273	-	-	0.000
**Herd size**					0.007
Large	0.769	0.274	2.159	1.262–3.693	0.005
Medium	0.263	0.305	1.300	0.715–2.365	0.390
**Husbandry system**					0.000
Intensive	0.766	0.227	2.151	1.380–3.355	0.001
Semi-intensive	1.083	0.231	2.954	1.878–4.645	0.000
Water courses	2.242	1.028	9.415	1.256–70.583	0.000

Note: Model-2 log-likelihood 1090.211.

*χ*^2^ goodness of fit = 12.449; *p* value = 0.132.

B, logistic regression coefficient; Std err, standard error; OR, odds ratio; CI, confidence interval.

* , Model *χ*^2^ = 108.8902 with 10 df.

### Herd level

The univariate herd level analysis revealed five factors associated with *T. evansi* seropositivity (Table 3): geographical location (*p* = 0.004), husbandry system (*p* = 0.038), history of abortion (*p* = 0.005), herd size (*p* = 0.010), and nearby natural water sources (*p* < 0.05; Table 3). *Trypanosoma evansi* seroprevalence was higher (*p* < 0.001) in Ghardaia, Biskra and El-Oued than in Ouargla. In contrast, no association was found between *T. evansi* seropositivity of the herd and contact with other camel herds, husbandry system (nomadic) or introduction of purchased camels (Table 3).

**TABLE 3:** Factors associated with animal-level seroprevalence of antibodies against *Trypanosoma evansi* for dromedary camel populations of southeastern Algeria.

Factor	Category	Number of herds	Serum *T. evansi* antibodies				p*
			Positive		Negative		
			*n*	%	*n*	%	
Geographic location (district)	Ouargla	21	9	42.9	12	57.1	0.004*
	Biskra	15	12	80.0	3	20.0	
	El-Oued	28	23	82.1	5	17.9	
	Ghardaia	18	16	88.9	2	11.1	
Contact with other camel herds	Yes	75	55	73.3	20	26.7	0.902
	No	7	5	71.4	2	28.6	
Husbandry system	Extensive	53	34	64.2	19	35.8	0.038*
	Semi-intensive	16	15	93.7	1	6.3	
	Intensive	13	11	84.6	2	15.4	
Divagation	Yes	36	28	77.8	8	22.2	0.405
	No	46	32	69.6	14	30.4	
History of abortion	Yes	27	25	92.6	2	7.4	0.005*
	No	55	35	63.6	20	36.4	
Introduction of purchased animals	Yes	44	33	75.0	11	25.0	0.687
	No	38	27	71.1	11	28.9	
Watering type	Artificial wells	43	26	60.5	17	39.5	0.006*
	Lakes and streams	39	34	87.2	5	12.8	
Contact with sheep and goats	Yes	32	23	71.9	9	28.1	0.832
	No	50	37	74.0	13	26.0	
Accessibility to natural water sources	Yes	50	41	82.0	9	18.0	0.024*
	No	32	19	59.4	13	40.6	
Educational level of farmer	Primary education	32	21	56.6	11	34.4	0.467
	Illiterate	41	32	78.0	9	22.0	
	University	9	7	77.8	2	22.2	

Note: An increase in herd size was associated with an increase in seropositivity (*p* < 0.001).

* , Univariate analyses (*χ*^2^ test for significance).

The multivariate logistic regression model showed that the main risk factor associated with *T. evansi* infection in camel herds was geographical location (Table 4). The likelihood of detecting *T. evansi* antibodies was 11 times higher in the district of Ghardaia (OR = 11.25; 95% CI [2.00–63.19]), 7.5 times higher in El-Oued (OR = 7.50; 95% CI [2.02–27.86]) and 6 times higher in Biskra (OR = 6.00; 95% CI [1.27–28.25]) than in the district of Ouargla (Table 4).

**TABLE 4:** Risk factors for herd-level infection with *Trypanosoma evansi* amongst dromedary camel populations in southeastern Algeria.

Category	B	Std err	OR	95% CI (OR)	p*
Constant	−0.405	0.456	-	-	0.374
Geographic location (district)	-	-	-	-	0.005
Ouargla	Reference	-	-	-	-
Biskra	1.792	0.791	6.000	1.274–28.254	0.023
El-Oued	2.015	0.670	7.500	2.019–27.862	0.003
Ghardaia	2.420	0.147	11.250	2.004–63.198	0.006

Note: Model-2 log-likelihood 81.281.

*χ*^2^ goodness of fit = 0.000; *p* value = 1.000.

B, logistic regression coefficient; Std err, standard error; OR, odds ratio; CI, confidence interval.

* , Model *χ*^2^ = 14.093 with 3 df.

## Discussion

To our knowledge, few studies have been performed in Algeria aimed at assessing risk factors associated with *T. evansi* infection of dromedary camels. Our results, which document high seroprevalence rates at the individual and herd levels, could ultimately guide the design and implementation of enhanced surveillance programmes, control measures and prevention strategies for *T. evansi* not only in eastern Algeria but throughout major camel producing regions of the world.

In Algeria, *T. evansi* infection in camels was confirmed for the first time more than a century ago when parasitological examinations discovered that 10% of 282 camels were infected (Sergent & Sergent 1905). Outbreaks of trypanosomosis in dromedary herds associated with mortalities and abortions have been documented (Boushaki et al. 2019). The *T. evansi* seropositivity rate in our study (49.5%) was higher than previous serological studies conducted in Algeria by Benfodil et al. (2020) and Boushaki et al. (2019), who reported seroprevalence up to 32.4%, and 9.9%, respectively. The higher seroprevalence in the present study could be because of the differences in the topography of the study areas, age of tested animals, proportion of males versus females, sample size, environmental conditions during the sampling period, or differences in diagnostic methods.

However, the *T. evansi* seroprevalence rate in our study (49.5%) is highly consistent with the findings from studies conducted in other developing countries that revealed *T. evansi* seroprevalence rates of 46% in Burkina Faso (Dia 2006), 45.9% in Kenya (Njiru et al. 2004), 43.8% in Saudi Arabia (El-Wathig & Faye 2013), 43.5% in Egypt (Abdel-Rady 2008) and 47.7% in Pakistan (Tehseen et al. 2015). One study in Somalia reported a higher *T. evansi* seroprevalence rate (56.4%) than that found in our study (Baumann & Zessin 1992). Thus, we interpret our results as being in line with results from several studies conducted in other camel-producing regions of the world.

Researchers in various African and Middle Eastern countries found lower *T. evansi* seroprevalence rates than ours. One study in Jordan found a 33% infection rate (Al-Rawashdeh et al. 1999) whilst in eastern Chad a 30.5% seroprevalence rate, detected using the CATT test, was reported (Delafosse & Doutoum 2004). Even lower rates of *T. evansi* seroprevalence were reported in the African nations of Ethiopia (between 18.2% and 24.9%; Aregawi et al. 2015; Bogale, Kelemework & Chanie 2012; Fikru et al. 2015; Hagos et al. 2009), Mauritania (24%, Dia et al. 1997), Niger (12%, Pacholek et al. 2000) and Tanzania (8.3%, Njiru et al. 2002). Others reports of low *T. evansi* seroprevalence came from UAE (10.67%, Chaudhary & Iqbal 2000), Iran (10%, Zarif-Fard & Hashemi-Fesharki 2000) and Pakistan (4%, Hasan et al. 2006). The heterogeneity between seroprevalence rates in different parts of the world is likely because of the differences in density of camel rearing, animal husbandry systems, climatic conditions, density of mechanical vectors, local herd management practices, study sample sizes, as well as cut-off values and sensitivity differences in the serological tests employed. Moreover, in our study location, poor-quality veterinary service, divagation and migration of camels to humid area and northland areas in search of feed (Benaissa et al. 2012) might have additionally contributed to the high seroprevalence. Under such production conditions, it will be important to educate camel farmers how to mitigate risks of *T. evansi* infection.

At the herd level, our study demonstrated wide distribution of camel trypanosomosis. Indeed, 73.2% of herds (60 of 82 tested herds) had at least one seropositive animal. Our overall herd seroprevalence is comparable to the 80% herd *T. evansi* seroprevalence reported recently in Somalia (Mohamed et al. 2020).

Our study documents a high prevalence of this disease-causing parasite and corroborates field observations of substantial waves of mortality and abortion reported by camel farmers and veterinarians in the region. The high herd-level seroprevalence supports field reports of marked financial impacts of this disease and indicates the need to develop field-practical control and prevention programmes.

Although few studies have addressed herd-level seroprevalence, our seropositivity result was much higher than the 42.3% reported earlier in Algeria (Boushaki 2007). Given the span of time between studies, our study highlights the continued circulation of the parasite within herds in the region as well as its endemicity. In uncontrolled situations, herd-level seroprevalence could reach as high as 94.9% (Delafosse & Doutoum 2004). In fact, two important risk factors identified at the individual level in the former study (contact between herds and introduction of new camels into the herd) support that infection can easily spread from one herd to the other.

No effect of camel age on *T. evansi* seropositivity was observed in our study. Our finding agrees with some surveys (Boushaki et al. 2019; Pathak & Khanna 1995; Shah et al. 2004), but differs from those of other research groups who reported increased seroprevalence with increasing age (Atarhouch et al. 2003; Bogale et al. 2012; Dia et al. 1997; Eshetu, Desta & Amare 2013; Gutierrez et al. 2000; Mirshekar, Yakhchali & Shariati-Sharifi 2017; Olani et al. 2016; Tehseen et al. 2015). In contrast to the above findings, one study in Ethiopia revealed higher infection rates in younger camels than in older adult camels (Lemecha, Lidetu & Hussein 2008). These conflicting results suggest that future investigations need to be designed to specifically test the hypothesis of age effects on *T. evansi* seropositivity.

The risk of contracting trypanosomosis in semi-intensive and intensive systems was approximately three and two times higher, respectively, than in extensive management systems in our study. This result agrees with previous reports that husbandry systems and practices have a great impact on circulation of *T. evansi* (Dia et al. 1997). Serological surveys conducted in Sudan detected a higher prevalence of *T. evansi* in nomadic camels than in camels managed in an agropastoral system (Elamin, El Bashir & Saeed 1998). Camel husbandry systems are frequently characterised by a remarkably high level of animal mobility and exchange between farms and areas, and even between countries (e.g., Algeria, Tunisia, Libya, Mali, and Niger). These highly common movements (controlled or uncontrolled) of camels could explain the wide distribution of *T. evansi* infection.

An association between camel seropositivity for *T. evansi* and herd history of abortion was observed in our study. This finding is in good agreement with a recent epidemiological study where abortion was associated with *T. evansi* seropositivity (Boushaki et al. 2019). This result was not unexpected, as abortions induced by *T. evansi* have been reported in other species. Unfortunately, data concerning the incidence of abortions and other productivity measures are frequently lacking in camel-dependent livestock production settings.

Herd *T. evansi* seropositivity rates varied greatly between geographical areas. The highest risk of camel trypanosomosis was observed in Ghardaia (a region with relatively cold climate). Ghardaia and Biskra, situated in the northern Algerian Sahara, have an abundance of naturally occurring water sources that are associated with higher rates of *T. evansi* seropositivity. Although our study area belongs to the arid climate stage, it remains rich in natural water resources, with several rivers as well as temporary lakes and wadis appearing during the rainy season. We interpret our results to mean that these natural water sources likely enhance the spread of trypanosomosis through the development and proliferation of tabanids, which are indiscriminate in the transmission of *T. evansi*. Because these vectors seem subservient to standing water (ponds and lakes), regions with wetlands and saline lakes can be identified as higher risk areas as reported recently in a study in seven others provinces of southern Algeria (Boushaki et al. 2019).

Worldwide, several authors have reported considerable differences in the seroprevalence of *T. evansi* in different geographical areas within the same country (Aregawi et al. 2015; Bogale et al., 2012; Delafosse & Doutoum 2004; Dia et al. 1997; Mirshekar et al. 2017; Olani et al. 2016; Salah, Robertson & Mohamed 2019; Tehseen et al. 2015). In most cases, these within country differences can be explained by the observed variation in ecological factors (e.g., pastoral camel densities, hottest, most humid and wettest districts in the region) that affect vector densities and therefore infection prevalence (Hagos et al. 2009). Interestingly, geographical variation was not considered in some recent animal-level studies of *Trypanosoma* seroprevalence (Ghaemi, Zavarib & Jannati Pirouz 2019; Hassan-Kadle et al. 2019; Salah et al. 2019). The geographical distribution of the parasite and vectors, and thus the occurrence of trypanosomosis, is largely dependent on the nature of the agro-ecological environment. Trypanosomosis, therefore, is regarded as endemic in certain regions (Holt et al. 2016). The climate is thought to permit and promote high densities of vectors such as the Tabanides fly (James et al. 1985).

Seropositivity for *T. evansi* infection was not associated with breed in our study. This result is in contrast with the studies that identified breed as a risk factor for *T. evansi* infection (e.g., in Chad; Delafosse & Doutoum 2004). It seems likely, however, that the confounding effects of breed and hair colour make interpretation of any potential breed effect difficult. Animals with dark hair (‘Zerga’) were three times more likely to be infected than camels with other colours of hair coat (Boushaki et al. 2019). Similarly, white-coloured camels in Saudi Arabia were more likely to be infected with *T. evansi* than animals with a dark coat (El-Wathig et al., 2016). Further complicating the interpretation of potential breed effects is the notion that various colour coat phenotypes may belong to the same genotypes (Almathen, Mwaracharo & Hanotte 2012; Cherifi et al. 2017). It should be noted that some other studies showed that camels were equally susceptible to *Trypanosoma* infection regardless of breed (Pathak & Khanna 1995).

No difference in *T. evansi* seropositivity was observed between male and female camels in our study. There are conflicting reports in the literature, however, regarding the effect of sex. Some studies reported that *Trypanosoma* infection rate was higher for females than males (Dia et al. 1997; Shah et al. 2004; Sobhy et al. 2017). Contrarily, one study in Mali (Ndoutamia et al. 1999) found higher rates of infection amongst males – a finding supported by a Kenyan study where *Trypanosoma* infection rates were 2.6 times higher in males than females (Njiru et al. 2004). These conflicting results may be explained by interactions with other factors, disproportionate sampling of males versus females or differential application of husbandry practices to male versus female camels. In many countries, male camels are housed in a pen or tied when females go to the rangelands for grazing. This differential management approach may explain apparent sex differences rather than there being an actual biological predisposition to infection linked to camel sex.

Serologic testing is an important method for detecting parasitic infections. The direct card agglutination test (CATT test) is a validated test that is considered a rapid tool of choice for detection of *T. evansi* infection in camels (Dia et al. 1997; Ngaira et al. 2003), buffalos (Davison et al. 1999) and cattle (Reid & Copeman 2003). The CATT test has been the test of choice for field investigations, despite the low sensitivity reported in some studies: 65.5% (Ngaira et al. 2003) and 68.6% (Njiru et al. 2004). The *T. evansi* direct card agglutination test (CATT) uses latex agglutination of beads coated with recombinant antigens RoTat 1.2 VSG (Manual 2012). Moreover, other reports (Benfodil et al. 2020; Diall et al. 1994; Gutierrez et al. 2000; Pathak et al. 1997) have shown a moderate to strong agreement between CATT and other diagnostic methods such as trypanosome lysis (TL), WCL ELISA and RoTat 1.2 type A ELISA. Recent validation studies on serologic tests for *T. evansi* infection in camels and other ruminants showed the degree of concordance was highest between TL and *T. evansi* CATT (*k* = 0.784), thus confirming the interest of using *T. evansi* CATT for serodiagnosis of surra in the field (Benfodil et al. 2020).

Because of its mechanical means of transmission, *T. evansi* may be disseminated through a wide range of vectors. The multitude of potential vectors allowed *Trypanosoma* to become the most widespread pathogens in tropical and subtropical areas (Hoare 1972). Although *T. evansi* is not pathogenic to humans, human infestation by this parasite was first reported in India (Joshi et al. 2005). A follow-up study (Shegokar et al. 2006) reported a 22.7% *T. evansi* seroprevalence in humans in India. This finding is probably the consequence of frequent exposure of humans to *T. evansi*-infected livestock.

Camel farmers can experience considerable economic losses because of *T. evansi* infections because they result in outbreaks of abortions (and subsequent loss of replacement breeding stock or meat animals), decreased milk production, loss of body condition, decline of production efficiency and increased mortality. Additional financial losses occur because of premature culling of sick animals and biannual treatments administered to *T. evansi* seropositive animals. These devastating financial impacts point out the need to establish effective surveillance, control and prevention programmes.

Conditions in which contact with a vector is enhanced, such as sedentary camels sharing a common water source in an oasis or contact between animals and target potential reservoirs, are common in camel rearing (Elamin et al. 1998). In the particular case of camel husbandry in Africa, nomadism is characterised by migration during the dry season to areas with bioclimatic areas or conditions favouring survival of the vectors (e.g., woodlands, valleys, permanent water). Nomadism, therefore, is an important risk factor (Baumann & Zessin, 1992; Delafosse & Doutoum, 2004; Dia et al. 1997; Ngaira et al. 2003).

In the present study, *T. evansi* seroprevalence in individual camels in large camel herds reached 52%, which corresponded to a risk of infection that was 2.1 times higher than camels from small herds. This higher risk is probably because of the fact that, in large farms, the infection pressure is stronger with more frequent contact between infected and non-infected animals. Our results agree with a study in Chad, which revealed that the largest herds were associated with a higher risk of trypanosomosis (Delafosse & Doutoum 2004).

The source of water (e.g., rivers versus artificial wells) was a major risk factor for exposure to *T. evansi* in our study. Herds whose primary water source was a river had greater rates of infection than herds that drank from wells. Also, the presence of a dense canopy and permanent ponds create ideal breeding conditions for various species of tabanids, thus promoting spread of the disease (Doutoum et al. 2002). Where feasible, camel farmers should reduce access to naturally occurring water sources and instead provide well water.

## Conclusion

Dromedary camels in eastern Algeria showed high rates of seropositivity for *T. evansi* at both the individual and herd levels. At the individual level, the three main risk factors for trypanosomosis were herd size, animal management system and accessibility to naturally occurring sources of water (rivers, lakes). Herd seroprevalence varied to a much lesser extent because of geographical location (district). These findings stress the importance of an integrated disease surveillance system for *T. evansi*. Preventive measures are needed to improve the health status of camel herds and to reduce the economic losses associated with outbreaks of camel trypanosomosis (which include abortions, cachexia, decreased productivity and mortality). Preventive measures may include surveillance of animal movements, particularly in rural areas. Studies employing more sensitive molecular diagnostic techniques, as well as epidemiological surveys to estimate seroprevalence at a national level, are needed to develop an efficient control strategy to avoid an endemic state of disease. In the context of global climate change, trypanosomiasis has been recognised as a re-emerging disease and a serious and economically important health issue with particular interest for Africa and Asia.
